# Epinephrine-reduced articaine solution (1:400,000) in paediatric dentistry: a multicentre non-interventional clinical trial

**DOI:** 10.1007/s40368-013-0024-9

**Published:** 2013-04-05

**Authors:** P. W. Kämmerer, N. Krämer, J. Esch, H. Pfau, U. Uhlemann, L. Piehlmeier, M. Daubländer

**Affiliations:** 1 Department of Oral and Maxillofacial Surgery, University Medical Centre, Mainz, Germany; 2 Policlinic for Paediatric Dentistry, University Gießen, Giessen, Germany; 3 Private Practice for Paediatric Dentistry, Munich, Germany; 4 Private Dental Practice, Herrsching, Germany; 5 Dental Surgery Outpatient Clinic, University Medical Centre, Mainz, Germany

**Keywords:** Paediatric, Dental, Local analgesia, Multicentre, Articaine, Epinephrine

## Abstract

**Aim:**

In paediatric dentistry, epinephrine may contribute to systemic and local side-effects. On the other hand it is necessary to provide good and safe local analgesia. Therefore, an articaine solution with reduced epinephrine concentration was tested in a clinical setting.

**Methods:**

In a non-interventional clinical study, dental treatment was performed in children and adolescents (4–17 years). For local analgesia, articaine 4 % plus epinephrine 1:400,000 was used in the technique chosen by the dentist. Efficacy and tolerance as well as duration of soft tissue analgesia and side-effects were evaluated.

**Results:**

999 patients (50.5 % male, 49.5 % female) with a mean age of 7.9 (SD 2.34) years were treated. Two hundred seventy six patients (27.6 %) received sedation prior to treatment. The mean treatment time was 15 min (SD 10). In 93.5 % of cases, initial local analgesia was sufficient to perform the planned treatment. In 99 % of cases (*n* = 989) the planned treatment could be completed. A second injection was necessary in 6.5 % of cases. A mean duration of soft tissue analgesia of 2.19 h (SD 1.01) was seen. Slight side-effects occurred in 3.1 % of subjects.

**Conclusions:**

Due to high efficacy, tolerance and safety, the articaine 4 % solution with the reduced epinephrine concentration (1:400,000) was a safe and suitable drug for paediatric routine treatment.

## Introduction

Local analgesia, which allows a virtually pain-free treatment, plays a crucial role in paediatric dental practice. Articaine is a commonly used local analgesic that was introduced to the German market in 1976. Experts have said that it may be the analgesic of choice in children over 4 years of age (Nizharadze et al. 2011). For younger children, a current meta-analysis could not find recommendations for its use, since no data supporting such were found (Katyal 2010). To avoid the risk of toxicity, especially when treating small children, a body weight-based dosage has to be calculated (Ahmed and Martinez 2009) and the use of a vasoconstrictor is recommended (Lipp et al. 1993; Meechan et al. 1994; Yagiela 1995). The vasoconstrictor of choice in most cases is epinephrine (Paxton and Thome 2010). For articaine, it was shown that adverse reactions occur mainly due to the amount of epinephrine in the analgesic solution (Santos et al. 2007). It may lead, especially at sites with increased resorption of the local analgesic or in cases of intravascular injection, to an increased heart rate, ejection volume, blood pressure, body temperature and blood supply of the skeletal muscles. Further systemic side-effects of the vasoconstrictor may be nausea, agitation, dizziness and tremor. A higher amount of epinephrine is equated with an increased analgesic duration (Kämmerer et al. 2011). As result of the longer loss of sensation after dental analgesia—especially in smaller children, self-induced lesions of the soft tissues such as biting of the tongue, the lips and the cheeks may occur (Ram and Amir 2006; Adewumi et al. 2008).

There are no special recommendations about the vasoconstrictor concentration for the treatment of children and it has been demonstrated that even a low concentration of epinephrine (1:400,000) leads to a significant prolongation of the systemic absorption of a local analgesic solution (Hansen et al. 2001). With a higher percentage of vasoconstrictor, the systemic as well as local risks may increase (Kämmerer et al. 2011). Altogether, a limitation of the amount of epinephrine in combination with articaine in paediatric patients should be discussed. An adaption of local analgesia to the respective treatment time may enhance the child’s wellbeing. Articaine, with the minor adjunct of epinephrine 1:400,000, has already been shown to be safe and effective in short dental procedures in adult patients (Daubländer et al. 2012). For children, there are no data available so far.

Therefore, the aim of this study was to conduct a non-interventional, multi-centre assessment of an articaine solution with reduced epinephrine concentration in dental paediatric practice. Efficacy and tolerance of the drug were primary criteria. Duration of soft tissue analgesia and possible side-effects were also evaluated.

## Methods

### Study design and patient selection

A prospective clinical study was performed at a Dental Clinic (University of Giessen) and four private dental practices for Paediatric or General Dentistry (Munich and Herrsching) between 2009 and 2010. Five dentists participated in the treatment. The study design was non-interventional (observational). A non-interventional study is defined as a study where the respective product is used in the usual manner and the assignment of the patient to a particular therapeutic strategy falls within current practice. This type of trial usually reflects normal clinical practice with a quality close to those from clinical and interventional trials (Worz and Hundt 2011). Accordingly, no calibration of the dentists was needed. The study was conducted with the approval of the Ethics Committee of the medical association of Rhineland-Palatinate and in accordance with the Declaration of Helsinki.

After taking the medical history of the patient, the procedures, possible discomforts or risks, as well as possible benefits were explained fully to the patients and their legal guardians. The legal guardians of the patients signed an informed consent prior to the initiation of the dental treatment. Inclusion criteria were: patients aged 4–17 years requiring routine dental treatment under local analgesia. Exclusion criteria were the following: contra-indications for one of the components of the analgesic solution (articaine, epinephrine, or sodium sulphite), limited activity of plasma cholinesterases, patients with American Society of Anaesthesiologists classification >2, lack of compliance and infections in the area of injection. If the respective criteria were met, each patient attending at one of the study centres for treatment was included in the study. The local analgesic solution was supplied in 1.7 ml carpules by 3 M ESPE (Seefeld, Germany) containing 4 % articaine plus epinephrine 1:400,000 (Ubistesin lite™ 1:400,000). It was used in the technique chosen by the dentist (infiltration, nerve block, periodontal ligament injection (PDL), combinations) with the instrument chosen by the dentist. Routine dental treatment (cavity preparations, extractions, endodontic treatment), was performed, mostly on primary teeth. The main indication for the epinephrine-reduced solution was for a shorter treatment time. Therefore, the expected treatment time should not extend 30 min. Additional sedation was performed if and when needed in accordance to the normal and everyday routine procedures of the clinic or practices. If needed, the dentist used nitrous oxide as an inhalation technique or orally administered midazolam and/or additional analgesics.

### Treatment protocol

Prior treatment, affected teeth, region as well as the respective indication were documented. The dentist made a decision concerning sedation and dose of the local analgesic. For each patient, a region of ≤3 adjacent teeth was examined. If there were more than one area to be treated, the region that was treated first was documented. For each patient, the duration of the treatment as well as the achieved analgesia (complete, sufficient, insufficient, none) after a period of 5–7 min was noted. If the analgesia was rated “insufficient” or “none”, a second injection was administered and documented. The patient assessed the efficacy subjectively. In addition, the injection technique as well as dosage of the local analgesic (primary and secondary injection as well as total dose) were documented. If sedation was needed, the character of the sedation as well as the special drug and its concentration were recorded. The dentist rated the quality of local analgesia after the treatment as “complete”, “sufficient”, “insufficient” and “no rating possible”. If “insufficient” or “no rating possible”, failed analgesia was documented. One day after treatment, a structured telephone interview with the patient or the parents was conducted to examine the duration of subjective soft tissue analgesia—recorded in minutes by the patients and/or legal guardians while touching the tissues—as well as potential side-effects. The patients remained on a follow-up schedule for 14 days after treatment.

### Statistics

All patients (respectively, legal guardians) that gave informed consent and were treated with Ubistesin™ 1:400,000 were evaluated in the study. A prior power calculation showed a sample size of *n* = 918 patients to be sufficient to reveal potential side-effects with an incidence of 0.5 % or more with a likeliness of 99 %. To find possible associations between side-effects and age and to obtain information about the influence of prior sedation on duration of soft tissue analgesia, students’ *t* tests were conducted. To assess an association between side-effects and injection technique as well as prior sedation, *χ* ^2^ tests were used. A difference between the groups was seen to be statistically significant if *p* < 0.05. To show a possible correlation between dose of local analgesic agent and soft tissue analgesia, Pearson’s correlation coefficient was calculated. All other data were descriptive only. For statistical evaluation, the Statistical Analysis System software (SAS, version 8.2; SAS Institute Inc., Cary, North Carolina, USA) was used.

## Results

### Patients

The study recruited 999 patients (50.5 % male, 49.5 % female) with a mean age of 7.9 ± 2.34 years that were treated between 04/2009 and 04/2010 in five study centres. Contrary to the treatment protocol, five children under 4 years of age (0.5 %) were treated as well. The patients had a mean weight of 29.5 ± 10.09 kg. Accordingly, 101 patients (10.1 %) weighed less than 20 kg, 496 patients (49.6 %) 20 to <30 kg, 333 patients (33.3 %) 30–45 kg and 69 patients (6.9 %) >45 kg. Concomitant diseases were known in 18 patients (1.8 %); (in detail: neurodermatitis *n* = 3, attention deficit hyperactivity disorder *n* = 2, asthma *n* = 2, thyroid hypofunction *n* = 2, diabetes type I *n* = 1, allergic coryza *n* = 1, sore throat *n* = 1, slight cough *n* = 1, purpura anaphylactoids *n* = 1, congenital heart disorder *n* = 1, mucopolysaccharidosis *n* = 1, cardiac dysrhythmia *n* = 1, photodermatosis *n* = 1). In all patients, no prior allergic reactions towards local analgesic agents were recorded. In nine patients (0.9 %), a prior pain medication (ibuprofen *n* = 6, paracetamol (acetaminophen) *n* = 2, talvosilen (codeine and paracetamol) *n* = 1) was self-prescribed.

### Indications of treatment

**Fig. 1:**
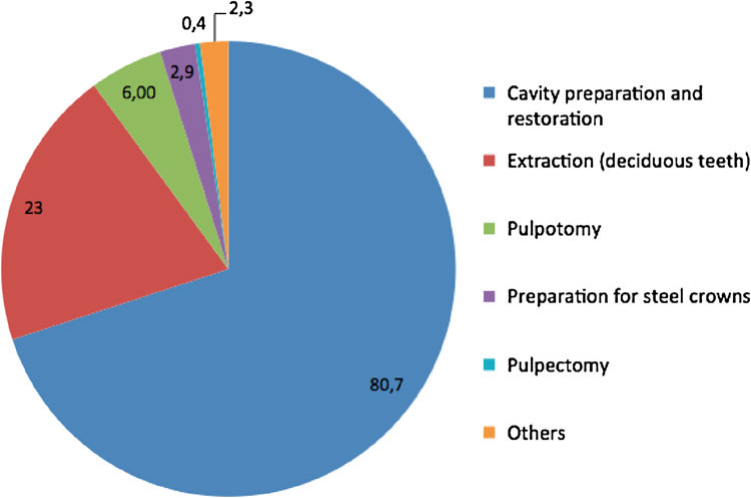
Pie chart showing the different indications for treatment in % (*n* = 999)

In 795 patients primary teeth and in 204 patients permanent teeth were treated. In 816 cases (81.7 %) dental cavity preparation and treatment with intra-coronal restorations were undertaken, in 230 cases (23 %) simple extractions of primary teeth and in 60 cases (6.4 %) endodontic treatments (pulpectomy, pulpotomy) on primary teeth were conducted. In 29 cases (2.9 %), teeth were prepared for preformed metal crowns. Other indicated treatment had a frequency of less than 3 % (Fig. 1). In 602 patients (60.3 %) one tooth, in 357 patients (35.7 %) two teeth and, in 40 patients (4 %) three teeth were treated.

### Analgesia

**Fig. 2:**
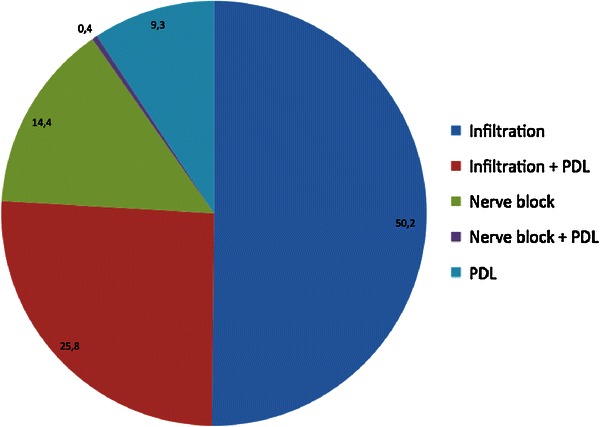
Pie chart showing the frequency of each injection technique in the study in % (*n* = 999)

Two hundred seventy six patients (27.6 %) received sedation prior to treatment [nitrous oxide *n* = 119 (11.9 %), midazolam + analgesics + other sedative *n* = 102 (10.2 %), midazolam + analgesic *n* = 31 (3.1 %)]. In 93.5 % of cases (*n* = 934) the initial local analgesia was complete or at least sufficient to perform the planned treatment. A second injection was necessary in 3.4 % (*n* = 34) before, in 2.7 % (n = 27) during treatment and in 0.4 % of cases (*n* = 4) at both times. The major technique used was infiltration (50.2 %, *n* = 501), followed by a combination with PDL (25.8 %, *n* = 258), block analgesia (14.3 %, *n* = 143), PDL only (9.3 %, *n* = 93) as well as block analgesia and PDL (0.4 %, *n* = 4) (Fig. 2). The mean volume of the initial injection was 1.1 ml (SD 0.43), the mean additional volume was 0.9 ml ± 0.46. The mean body weight-based dosage was 1.5 ± 0.69 mg/kg; in very young and frail children an increase up to 3.43 mg/kg was documented. Between first injection and start of treatment a mean time of 6 ± 4 min elapsed, and between first injection and second injection a mean time of 16 ± 9 min was measured. The mean treatment time was 15 ± 10 min.

**Fig. 3:**
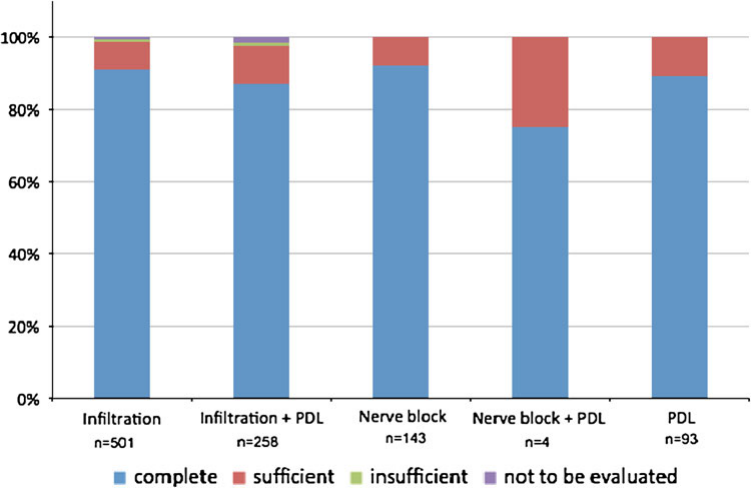
Bar charts showing the quality of local analgesia rated by the dentist. A subdivision into the different injection techniques is given

In 99 % of cases (*n* = 989) the planned treatment could be completed. Only 1 % of the patients (*n* = 10) were either non-compliant (*n* = 7) or very anxious (*n* = 1) or had insufficient response to repeated local analgesia (*n* = 1) or sedation (*n* = 1). In 98.7 % of cases, the quality of local analgesia was rated to be at least sufficient (Fig. 3).

### Side-effects

Fifty five unwanted side-effects occurred in 42 (4.3 %) of patients. A possible or likely coherence with the solution was stated in 3.1 % (*n* = 31). Of these, 71 % were light and all were transient. The main side-effects were unspecific systemic [nausea (1.1 %, *n* = 11), exhaustion (0.9 %, *n* = 9) and headache (0.5 %, *n* = 5)]. Post-operative soft tissue injury occurred in four patients (0.4 %); all were younger than 7 years (mean 5.4 ± 1.2). Erythema was reported in three cases (0.3 %). Other side-effects were seen in ≤0.1 % of cases. Side-effects were evaluated significantly more often in the group of children younger than 6 years of age (*p* = 0.001). Neither an association between side-effects and injection technique (*p* = 0.53) nor between side-effects and prior sedation (*p* = 0.78) was observed.

### Soft tissue analgesia

**Fig. 4:**
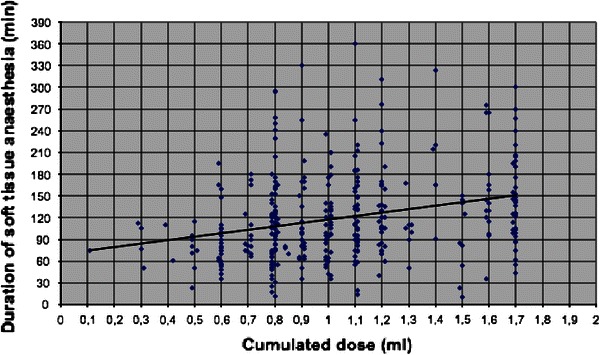
Scatter plot showing the weak correlation between the amount of local analgesic solution and the duration of soft tissue analgesia after infiltration

For *n* = 997 patients (drop-out rate was 0.2 %), a mean duration of soft tissue analgesia of 2.19 ± 1.01 h (total time from injection to end of numbness) was measured. The longest duration was seen for block analgesia with 2.43 ± 0.55 h, the shortest for PDL with 1.22 ± 0.47 h only. It could be shown that for infiltration, the duration of soft tissue analgesia tended to ascend with increasing volume of the local analgesic solution (Pearson’s correlation coefficient 0.26; Fig. 4). For the other techniques no such correlation was observed. Sedation increased the duration of analgesia in a small amount for all techniques, statistically relevant only in the infiltration plus PDL group (*p* = 0.049).

## Discussion

In paediatric dentistry, local analgesia offers virtually pain-free treatment, providing comfort for children and increasing their cooperation. To reduce plasma levels of the local analgesic and to enhance the analgesic effect, the use of a vasoconstrictor is recommended (Lipp et al. 1993; Meechan et al. 1994; Yagiela 1995), although, the vasoconstrictor may produce its own adverse side-effects (Meechan et al. 2001; Santos et al. 2007). To minimise those and to balance risk and benefit, a reduction of the vasoconstrictor in paediatric dentistry may be of value. Therefore, the aim of this non-intervention clinical study was an evaluation of efficacy, tolerance and safety of 4 % articaine with an adjunct of 1:400,000 epinephrine in dental treatment of children aged 4–17 years. For adult patients, our group could prove in a similar clinical setting that epinephrine-reduced articaine is safe and effective in short dental treatments (Daubländer et al. 2012). Though, to the best of our knowledge, this is the first study evaluating 4 % articaine with 1.400,000 epinephrine in paediatric dentistry. With a primary analgesia success rate of 93.5 % of cases, 99 % of all treatments were completed, although no self-reporting by the patients was included in the analysis. The latency of analgesia had a mean of 6 min. For latency with 4 % articaine with 1.200,000 epinephrine, a shorter time has been reported (Ram and Amir 2006). The influence of the vasoconstrictor concentration on analgesic diffusion due to a slower absorption rate may explain this difference (Lima-Junior et al. 2009; Kämmerer et al. 2012), although, this effect was not seen in infiltration analgesia (Moore et al. 2006). The volumes used in this study (mean = 1.1 ml) were generally very low; however, in smaller children, dosages up to 3.46 mg/kg of articaine were recorded. Those could be potentially dangerous when using a local analgesic without vasoconstrictor (Lipp et al. 1993; Meechan et al. 1994; Yagiela 1995). Altogether, we could demonstrate that with the low concentration of the vasoconstrictor used in the present study, efficacy as well as mean duration of analgesia was sufficient in nearly all cases. Efficacy and safety of 4 % articaine with the higher epinephrine concentrations of 1:100,000 and 1:200,000 in dental treatment of children have been studied before; showing a safe and efficient effect of the respective solution. There was also no higher incidence of adverse reactions following 4 % articaine with epinephrine 1:100,000 in children under the age of 4 years, although the manufacturer does not recommend this use (Wright et al. 1981, 1989; Dudkiewicz et al. 1987). A study on the pharmacokinetics of articaine with epinephrine 1:200,000 in children (3–12 years old) showed drug serum levels comparable to adults and no relevant differences between the 2 and 4 % solution. The maximum plasma levels were distinctly earlier and the plasma clearance increased in comparison to adult subjects (Jakobs et al. 1995).

A major disadvantage of local analgesia in children is the prolonged numbness after treatment, which may increase the chance of self-inflicted soft tissue lesions. The effect of numbness has been reported to be longer after articaine use than other local analgesics such as lidocaine (Ram and Amir 2006). As described by Adewumi et al. (2008), younger children especially may primarily experience such side-effects. Nevertheless, due to the small number of such side-effects in the present study, this result may be seen controversial. We recommend further trials with narrower age limits, basing the studies on the younger age group.

Within the limitations of the present study (phone call 24 h later with an increased chance of recall bias), the mean duration of soft tissue analgesia was 2.19 h. Compared to adults using the same epinephrine-reduced articaine solution, the duration was approximately 20 min shorter in the paediatric population (Daubländer et al. 2012). Ram and Amir (2006) reported for 4 % articaine with epinephrine 1:200,000 a mean duration of total soft tissue analgesia of 3.43 h. Accordingly, the reduction of vasoconstrictor results in a shorter time of numbness. This may explain the smaller number of soft tissue injuries in our study (0.4 %) compared to the 14 % reported by Adewumi et al. (2008). Similar to prior studies, the duration of soft tissue numbness for local infiltration was shorter than the duration of nerve block injections (Malamed et al. 2000). We also found a weak but clinical relevant correlation with the injected volume when using infiltration.

After administration of 4 % articaine with different epinephrine concentrations (1:100,000 and 1:200,000), no severe adverse effects in children were observed. Both solutions were shown to be efficient and safe (Dudkiewicz et al. 1987; Wright et al. 1989). Therefore, our data concerning the safety of 4 % articaine with 1:400,000 epinephrine tested in 999 children with a low rate of minor side-effects reflect the already known risk–benefit profile of articaine solution.

## Conclusions

Due to a high efficacy, tolerance and safety, articaine 4 % solution with reduced epinephrine concentration (1:400,000) is a safe and suitable drug in paediatric dentistry for routine treatment. For longer and very painful procedures and in treatments that require ischaemia, solutions with higher concentrations of epinephrine are preferable.
